# Assessment of Corrosion and Cavitation Resistance of Laser Remelted GX40CrNiSi25-20 Cast Stainless Steel

**DOI:** 10.3390/ma17246278

**Published:** 2024-12-22

**Authors:** Ion Mitelea, Ilare Bordeașu, Daniela Cosma, Dragoș Buzdugan, Corneliu Marius Crăciunescu, Ion Dragoș Uțu

**Affiliations:** 1 Department of Materials and Fabrication Engineering, Politehnica University Timisoara, Bulevardul Mihai Viteazul nr.1, 300222 Timișoara, Romania; ion.mitelea@upt.ro (I.M.); daniela.alexa@fih.upt.ro (D.C.); dragos.buzdugan@upt.ro (D.B.); corneliu.craciunescu@upt.ro (C.M.C.); 2 Department of Mechanical Machines, Equipment and Transports, Politehnica University Timisoara, Bulevardul Mihai Viteazul nr.1, 300222 Timisoara, Romania; ilare.bordeasu@upt.ro

**Keywords:** cast stainless steel, Yb-YAG laser remelting, erosion cavitation, corrosion

## Abstract

This paper explores the enhancement of cavitation and corrosion resistance in cast stainless steel through laser beam surface remelting. The influence of laser treatment on material properties was assessed by analyzing the microstructure using optical microscopy, electron microscopy, and X-ray diffraction. Cavitation erosion was evaluated in tap water using an ultrasonic vibration setup, following ASTM G32—2016 standards. Results show that local remelting of the surface with a laser beam causes a reduction in material loss and cavitation erosion rate. Potentiodynamic polarization tests revealed a significant improvement in corrosion resistance, indicated by a reduced corrosion current density in the laser-treated surface. The observed improvements in cavitation and corrosion resistance are attributed to microstructural hardening, characterized by grain refinement and a uniform, homogeneous structure with finely dispersed, small precipitate particles.

## 1. Introduction

The phenomenon of cavitation occurs due to the rapid reduction of pressure in certain zones within a liquid medium, followed by an equally abrupt increase in pressure. This decrease in static pressure below the vaporization limit leads to the formation of vapor-filled bubbles and gases. When subjected to high pressures, these bubbles implode or explode, generating shockwaves and high-speed microjets (on the order of hundreds of meters per second) that exert enormous pressures, reaching hundreds of bars on tiny surfaces. The repeated implosions create cyclic fatigue stresses, resulting in a specific type of wear known as cavitation erosion on the solid surface located in the area where this hydrodynamic phenomenon occurs [1,2,3,4,5,6].

The degree of cavitation erosion can be shown by the change in the mean erosion rate (the amount of mass loss per unit of time). There are four distinct stages that can be identified according to ASTM G32 standards:

- An incubation period, during which there are no measurable material losses, and the material undergoes elastic or plastic deformations, potentially forming some microcracks;
- An accumulation zone associated with an increase in the rate of material loss due to the propagation of cracks in the mechanically worked material;
- A mitigation region where the rate of mass loss is decreasing;
- A stable equilibrium state where the erosion rate is constant.

Due to their low resistance to cavitation erosion, materials used in components of hydromechanical systems subjected to local pressure fluctuations experience significantly shortened lifespans [7,8,9]. Microjets and shockwaves induce elastic and plastic deformations, mechanical hardening, formation of macles, cracking, and even structural damage by creating cavities [10,11,12,13].

So far, there have been multiple unsuccessful or only partially successful attempts to correlate cavitation erosion resistance with a single mechanical property or a combination of mechanical properties of metallic materials [1]. These mechanical properties include ductility characteristics, hardness, tensile strength, yield strength, resilience (KCU), fracture energy (KV), and the product of the fatigue strength coefficient and the cyclic strain hardening exponent [1,3,4,9]. However, these relationships are empirical in nature and provide predictions only for a limited group of materials.

Moreover, when the cavitation fluid is corrosive, material loss is not purely mechanical, as the corrosive factor also comes into play. In such cases, erosion induced by corrosion and/or corrosion induced by erosion will intensify the degradation process [1,3]. Erosion and corrosion often act synergistically, and material loss can be significantly higher than the sum of the effects of these processes acting separately [3,4,14,15,16,17,18,19,20].

The initiation and propagation of cavitation erosion are significantly influenced by the crystal lattice type of metallic materials. For metals with a face-centered cubic (f.c.c.) lattice, such as Ni, Cu, Al, and pure austenitic steels, cavitation cracks predominantly localize along grain boundaries and annealed twins, with the degradation mechanism being ductile rupture [8,9]. In the case of metals with a body-centered cubic (b.c.c.) crystal lattice (Cr, Mo, superferritic stainless steels), preferred sites for damage initiation consist of slip bands and non-metallic inclusions [10]. For metals with a hexagonal close-packed (h.c.p.) crystal lattice, such as Ti α, Mg, and Zn, boundaries between grains with an angle of misorientation greater than 42° and triple junctions exhibit low cavitation resistance [11]. Metallic materials with a biphasic microstructure, characterized by uniformity and a high degree of fineness, demonstrate higher resistance to cavitation degradation [12].

Improved maintenance results can be achieved through: a. careful selection of the materials used; b. application of surface modification processes (thermal spraying, hardfacing through welding, mechanical work hardening, Chemical Vapor Deposition CVD and Physical Vapor Deposition PVD, local remelting using TIG, laser, electron beam, etc.).

In addition to the mechanical effects of cavitation (mechanical erosion), metallic materials in contact with water can sometimes undergo chemical corrosion [16]. Various methods are employed to enhance resistance to cavitation and corrosion degradation, including the application of coatings through processes such as electric arc welding, thermal spraying, nitriding treatments in gaseous or plasma environments, and local TIG or laser remelting of the surface [21,22,23,24].

L.L. Silveira et al. [21] conducted a comparative analysis of the corrosion and cavitation resistance of FeCrMnSiNi and FeCrMnSiB layers deposited by High Velocity Oxygen Fuel HVOF and High Velocity Air Fuel HVAF spraying processes. They found that HVAF coatings exhibited lower levels of porosity and oxides, as well as higher hardness values, contributing to better resistance to cavitation and corrosion. S. Zhang et al. [22] demonstrated that plasma arc welding deposition of Colmonoy 88 alloy layers on an austenitic stainless steel substrate significantly increased resistance to cavitation and corrosion, attributed to the in situ-synthesized WC particles providing an anchoring effect on solid solutions to inhibit crack nucleation and propagation.

A.N. Allenstein et al. [24] highlighted an increase in cavitation erosion resistance for low-temperature plasma-nitrided martensitic stainless steel CA-6NM. This improvement was attributed to the formation of expanded nitrogen austenite (γN-Fe) from a tempered martensitic matrix.

The technique of local surface modification through laser beam remelting has been applied to numerous ferrous and non-ferrous metal alloys [25,26,27,28,29,30]. It offers several advantages, including the formation of a fine and homogeneous microstructure, ensuring a strong metallurgical bond between the surface layer and the substrate, and a minimally heat-affected zone.

C.T. Kwok et al. [25,28] demonstrated that continuous wave Nd:YAG laser melting of the surfaces of martensitic stainless steels improves the resistance to cavitation erosion and corrosion characteristics, particularly in pitting corrosion, attributed to the dissolution or refinement of carbide particles and the presence of residual austenite. C.H. Tang et al. [29,30] studied the influence of laser melting on the surfaces of special bronzes on cavitation and corrosion resistance, finding significant increases in cavitation erosion resistance attributed to an increase in surface hardness and a more homogeneous microstructure.

The laser local surface remelting process is a potential choice for addressing issues caused by material losses due to cavitation erosion and electrochemical corrosion. Employing this process is anticipated to achieve homogenization and refinement of the microstructure, as well as the dissolution or redistribution of precipitates or inclusions while preserving the properties of the substrate [25,27,29,30].

The GX40CrNiSi25-20 steel is widely used in systems handling cavitation and corrosive fluids, including applications in the automotive industry (turbochargers, manifolds, valves) and the construction of installations (for oil and gas, valve bodies). Its resistance to cavitation-corrosion is relatively low, limiting its use in such environments. To extend its application range, a feasible approach employed by the authors in this work is the use of local laser surface remelting as a modification method.

To the authors’ knowledge, the literature does not provide information on the influence of microstructural variation promoted by local laser remelting on the response of stainless steels with more than 0.1% C to cavitation and electrochemical corrosion erosion. Many such studies refer to stainless steels with lower carbon content (0.03–0.1% C). Cast stainless steels generally have corrosion resistance similar to their wrought counterparts, but they can become less resistant to cavitation erosion due to localized contamination, microsegregation, or lack of homogeneity.

This work aims to use the laser process as a heat source to modify the surface of stainless steel through local remelting, thereby improving hardness and resistance to cavitation and corrosion erosion.

## 2. Materials, Experimental Details

GX40CrNiSi25-20 (W.1.4848) steel, as per EN 10295, is a cast steel with numerous potential applications in fields such as aerospace, automotive, nuclear reactors, marine and river vessels, civil engineering components, and more.

Cylindrical specimens made of this steel, with the chemical composition provided, underwent a series of treatments to prepare them for further experimentation. Initially, the samples were cut to the dimensions specified (Ø 25 mm × 40 mm). Using a Thermo Scientific Arl QuantoDesk device from Burladingen, Germany, optical emission spectrometry was used to determine the steel’s chemical composition, which includes carbon (C), chromium (Cr), nickel (Ni), silicon (Si), manganese (Mn), molybdenum (Mo), phosphorus (P), and sulfur (S). The results are consistent with the supplier (Table 1). Chromium and nickel are the primary alloying elements that determine the structure, mechanical properties, and corrosion resistance of cast components made from this steel. The higher carbon content enhances mechanical strength characteristics but reduces ductility and toughness. Additionally, carbon can negatively affect corrosion resistance when it combines with chromium to form chromium carbides along grain boundaries.

**Figure 1:**
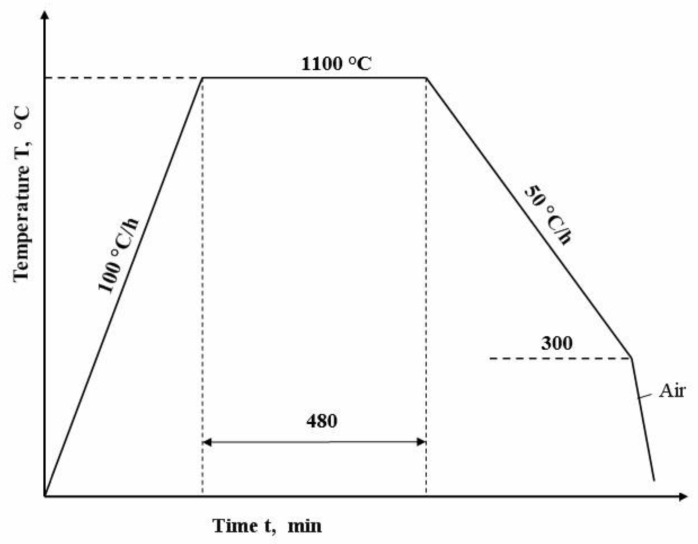
Heat treatment cyclogram.

Subsequently, the samples underwent a homogeneous annealing heat treatment process at a temperature of T = 1100 °C for 8 h maintaining time (Figure 1).

This annealing process was crucial for achieving a uniform microstructure throughout the samples and relieving any internal stresses that may have been present. After annealing, the samples were slowly cooled in the oven to ensure controlled cooling rates, which is important for the formation of desired microstructural characteristics.

**Figure 2:**
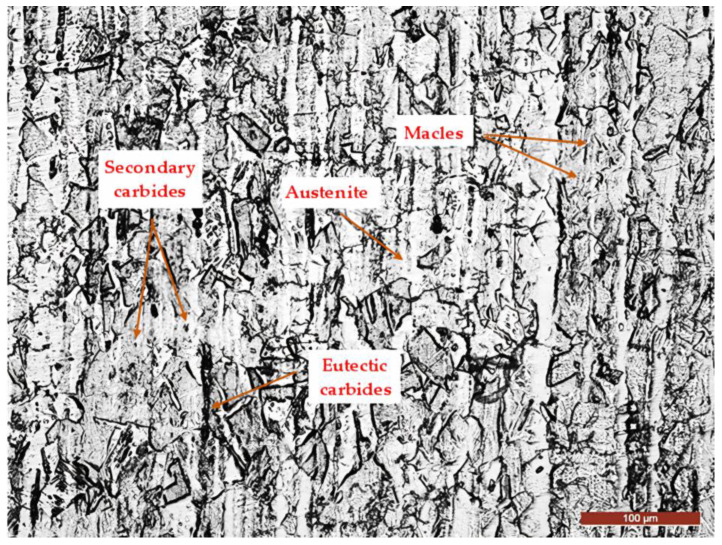
The microstructure of the investigated steel (Villella’s reagent)—MO × 200.

The microstructure of the steel, as observed in Figure 2, consists of austenitic grains with macles (twins), as well as eutectic and secondary carbides arranged along grain boundaries and in the direction of solidification dendrites. These observations are consistent with the findings of previous research studies [31,32], confirming the expected microstructural characteristics of the steel under investigation.

The modification of the thermal conditions for some of the samples involved laser remelting, which was carried out at three different energy densities. The energy density, denoted as E, is calculated using the formula:

(1) $$E=\frac{P}{v\cdot d},\frac{J}{{mm}^{2}}$$

where

P represents the laser power in kilowatts (kW),

v is the laser scanning speed (mm/min), and

d is the diameter of the laser spot (mm).

This equation illustrates how the energy density is affected by laser power, scanning speed, and spot diameter. Variations in these parameters influence the laser remelting process, consequently impacting the results obtained on the thermally treated samples.

**Figure 3:**
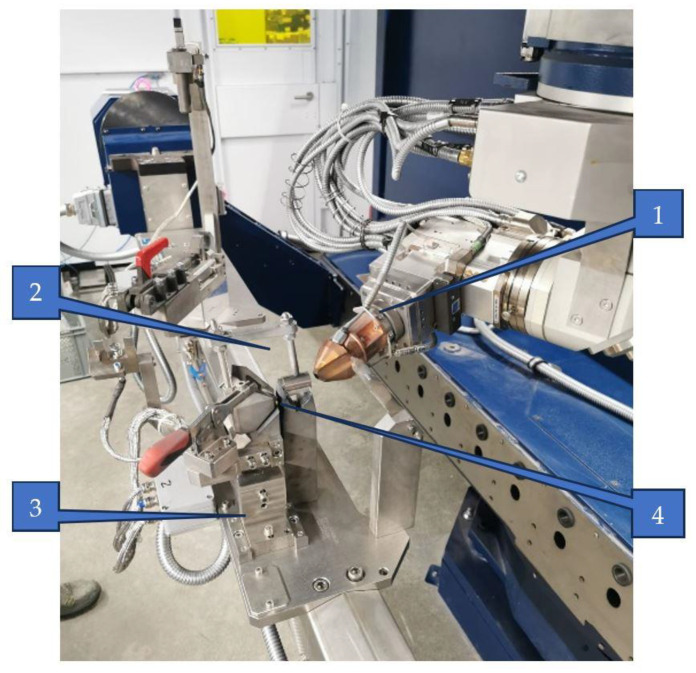
Sample setup: 1—Optical module; 2—Copper nozzle with central hole for laser beam and protective gas.; 3—Device; 4—Sample.

For the surface remelting process, a laser beam generated by the ytterbium system, specifically the Yb-YAG system, was employed, as depicted in Figure 3.

The equipment utilized for the surface remelting process is a Trumpf Trucell 7040, powered by the Yb-YAG laser source Trudisk 4002 (from TRUMPF Laser SE, Schramberg, Germany). This system emits a high-quality laser beam with a quality of 8 mm·mrad and a wavelength of 1030 nm. The laser beam is transmitted to the workpiece through a Brightline optical fiber with a diameter of 200 µm. This application was programmed using the Trutops software, version 13.0 that Trumpf offered.

Two numerically controlled linear axes hold the optical module that projects the laser beam onto the workpiece. It has three focusing, collimating, and reflective mirrors positioned on one translation axis, two additional rotation axes, and one more rotation axis.

Argon gas was used as a protective shield, with a flow rate of 10 L/min. A 16 mm gap was established between the nozzle and the workpiece material’s surface. The changeable parameter was the laser scanning speed, which varied between 800, 1000, and 1200 mm/min. The melted material traces varied in depth from 0.4 mm to 0.6 mm and in width from 0.9 mm to 1 mm, with a 50% overlap.

Once the programming was completed and the sample was securely positioned in the device, the actual melting process was initiated and fully automated. At a frequency of 5010 Hz and a power of 1000 W, the laser beam was turned on when the optical module had reached its initial position, being projected onto the workpiece with a pinpoint projection diameter of 1 mm.

**Figure 4:**
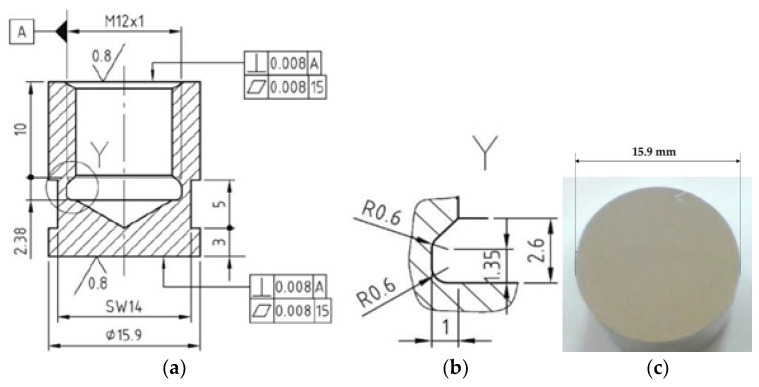
Cavitation test: execution specifications (**a**) axial slice via the specimen, (**b**) Y area detail (thread clearance), and (**c**) sample surface picture taken before the cavitation exposure.

The samples underwent surface remelting and bulk heat treatment, after which they were ready for cavitation (Figure 4) and electrochemical corrosion testing.

Cavitation tests were carried out on sets of three samples, where mass losses and erosion rates were measured over the cavitation attack exposure. The tests were conducted using an ultrasonic apparatus [33], following the guidelines outlined in the ASTM G32—2016 standard [34].

The experimental setup for the cavitation tests involved the following parameters and procedures:

- Ultrasonic Generator Settings: The ultrasonic generator was set to produce vibrations with a double amplitude of 50 μm and a frequency of 20.000 ± 2% Hz. An electrical power of 500 W was supplied to the generator to generate the required ultrasonic vibrations;
- Cavitating Liquid: Water from the public network was used as the cavitating liquid. The temperature of the water was maintained at 22 ± 1 °C throughout the experiment;
- Sample Preparation: Before subjecting the samples to cavitation attack, their surfaces were polished to achieve a specified roughness of Rz = 0.2 ÷ 0.8 µm. This ensured uniformity in surface conditions for all samples;
- Measurement Procedure: Each sample was weighed at regular time intervals using a Zatklady analytical balance with a precision of 10–5 g. This allowed for the measurement of mass loss due to cavitation erosion over time;
- Test Duration and Periods: The cavitation test duration was set to 165 min. This duration was divided into 12 periods (∆t), with each period consisting of intervals of 5 min, 10 min, and 15 min. This division allowed for monitoring the progression of cavitation erosion at different time intervals;
- Computer-Controlled Testing: The entire testing program was conducted using specialized software developed for this purpose, which enabled precise control and monitoring of the experimental parameters and data acquisition.

By employing this controlled experimental setup, it was possible to systematically study the effects of cavitation erosion on the samples under defined conditions, facilitating accurate measurement and analysis of erosion rates and material degradation over time.

The process for determining the corrosion behavior of the steel, both before and after laser beam remelting, involved the electrochemical method and subsequent microstructural analysis:

- Electrochemical Testing: A corrosion cell was utilized for the experimentation, consisting of the SP-150 galvanostat (Biologic, Seyssinet-Pariset, France). Three electrodes were used in the cell: the reference electrode (calomel), the auxiliary electrode (platinum electrode), and the working electrode (sample). For testing, 20 mm in diameter and 5 mm in thickness disc-shaped samples were made. These samples were ground and mirror-polished on the testing surface to provide a uniform and smooth surface finish. After preparation, the samples were degreased and ultrasonically cleaned in ethanol for 15 min to remove any contaminants or residues. The tested surface, which was in contact with the corrosive media, was 1 cm^2^. Using a rate of 0.166 mV/s, the applied potential was adjusted between −2500 and 2500 mV before the potentiodynamic polarisation tests were conducted. To guarantee a steady state, the samples were left at their open circuit potential for 30 min. All electrochemical measurements were performed at least three times to guarantee the results’ repeatability. Polarisation curves were drawn in a 3.5% NaCl and 0.5 M H_2_SO_4_ solution at ambient temperature. By using both corrosive media, researchers can compare how a material performs under different corrosive conditions: one simulating saltwater exposure and the other testing resistance to acid corrosion. This helps obtain a comprehensive understanding of the material’s corrosion behavior in various real-world environments;
- Microstructural Analysis: Cross-sectional slices of the samples, which were subjected to both cavitation and corrosion testing, were prepared for microstructural studies. The microstructure of the samples was examined using optical microscopy (Leica DM 2700 M) and scanning electron microscopy (TESCAN VEGA 3 LMU) with Bruker EDX Quantax. These techniques allowed for the visualization of the internal structure and any changes induced by cavitation and corrosion;
- X-ray diffraction analysis (Philips X’Pert Diffractometer, PANalytical, The Netherlands) was performed using Cu Kα radiation generated at 40 kV and 30 mA to identify the phases present in the structure;
- Surface Hardness Measurement: Surface hardness was measured using the Vickers HVS-10A1 instrument (from ZwickRoell, Ulm, Germany) at a load of 5 daN and a dwell time of 15 s. This provided information about the mechanical properties of the samples and any changes resulting from the laser remelting process.

By conducting electrochemical testing and microstructural analysis, it was possible to evaluate the corrosion resistance of the steel before and after laser beam remelting, as well as assess changes in microstructure and surface hardness. These analyses provided valuable insights into the effectiveness of the laser remelting process in improving the material’s resistance to both corrosion and mechanical degradation.

## 3. Discussing the Experimental Results

### 3.1. Cavitation Curves

**Figure 5:**
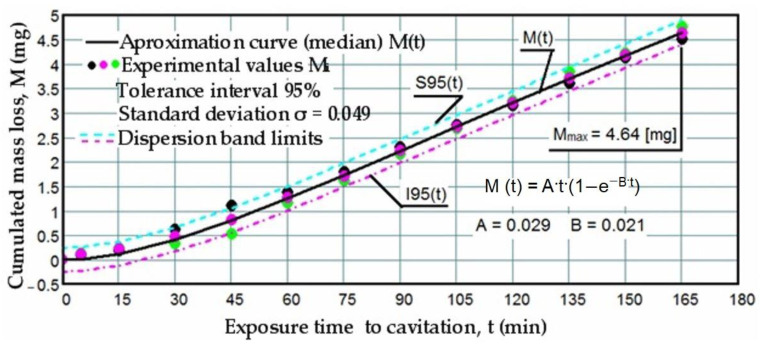
Variation of cumulative mass losses with cavitation time (v = 0.8 m/min, P = 1 kW, d = 1000 µm).

**Figure 6:**
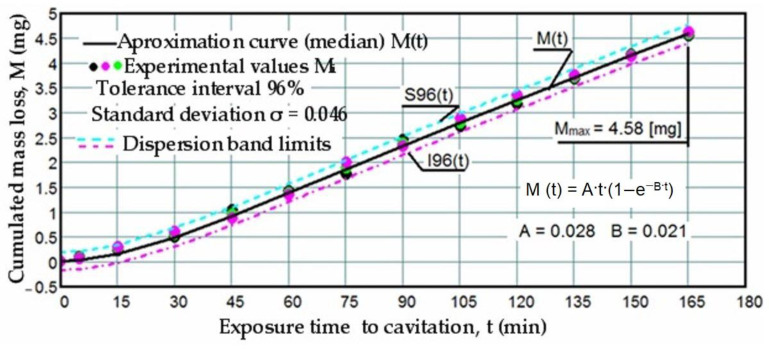
Variation of cumulative mass losses with cavitation time (v = 1.0 m/min, P = 1 kW, d = 1000 µm).

**Figure 7:**
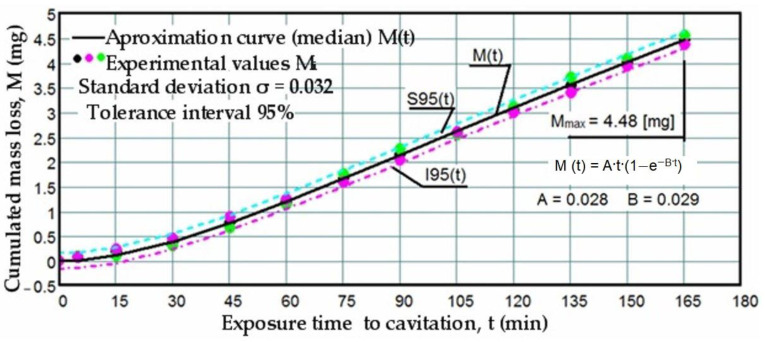
Variation of cumulative mass losses with cavitation time (v = 1.2 m/min, P = 1 kW, d = 1000 µm).

**Figure 8:**
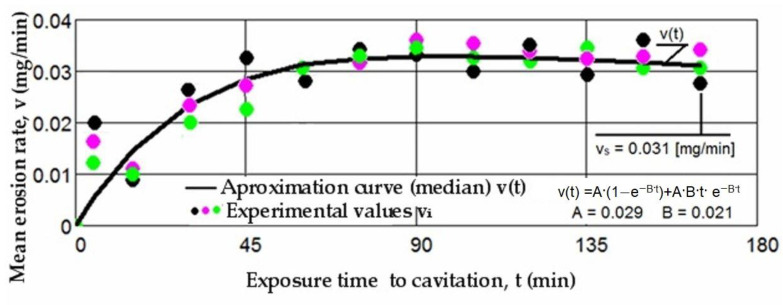
The variation of the mean erosion rate with the exposure time to cavitation (v = 0.8 m/min, P = 1 kW, d = 1000 µm).

**Figure 9:**
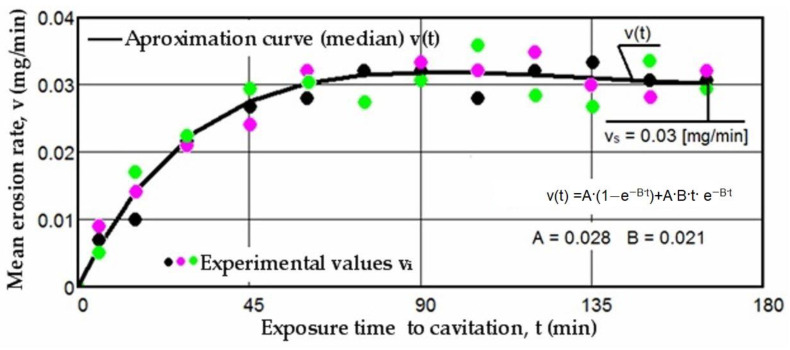
The variation of the mean erosion rate with the exposure time to cavitation (v = 1.0 m/min, P = 1 kW, d = 1000 µm).

**Figure 10:**
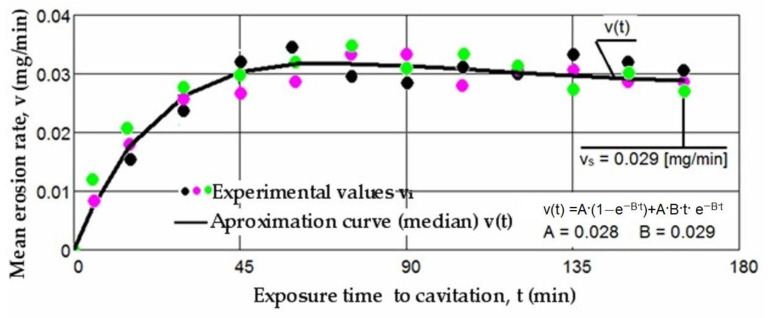
The variation of the mean erosion rate with the exposure time to cavitation (v = 1.2 m/min, P = 1 kW, d = 1000 µm).

Based on the mass losses determined with the analytical balance, diagrams were constructed showing the variation of the cumulative mass lost through erosion with the exposure duration to cavitation, as well as the corresponding erosion rates for the three laser scanning speeds. These Figures (Figure 5, Figure 6, Figure 7, Figure 8, Figure 9 and Figure 10) illustrate the curves representing the experimental values, which were constructed using laboratory-established relations [35,36], with the following forms:

M(t) = A·t·(1 − e^−B·t^) (2)

v(t) = A·(1 − e^−B·t^) + A·B·t·e^−B·t^

The experimentally determined values for the cumulative mass and erosion rate for each of the three samples in the set were introduced into the relations:

(3) $$M_{i}=\sum_{i=1}^{12}\Delta m_{i}$$

$$v_{i}=\frac{\Delta m_{i}}{\Delta t_{i}}$$

where

- M_i_—is the experimental value of the cumulative eroded mass lost over the time t_i_ = ∑Δt_i_ of the cavitation attack;
- Δm_i_—is the mass of material lost through cavitation erosion during the intermediate period “i” of time, Δt_i_ (for i = 1 time is Δt_1_ = 5 min, for i = 2 times is Δt_2_ = 15 min, and from i = 3 to i = 12 times, Δt_i_ increases by 15 min each period).
- v_i_—is the rate value corresponding to the mass loss during the period “i”.

To certify the accuracy of the experiment’s execution, upper (S) and lower (I) boundaries were delimited against the median curves M(t) based on the mean standard deviation σ in Figure 5, Figure 6 and Figure 7. This process formed the range of dispersion for the experimental points, referred to as the tolerance interval. The experiment utilized specific statistical relations implemented with the assistance of the Mathcad program.

The relations used to determine the mean standard deviation σ and the upper/lower limits S and I have the following forms [36]:

For the mean standard deviation:

(4) $$\sigma ={\left[\frac{\sum_{i=0}^{12}{\left(M_{i}-M(t{)}_{i}\right)}^{2}}{n-1}\right]}^{\frac{1}{2}}$$

for the 99% tolerance interval:

S99(t) = MDE(t) + 1 · σ;  I99(t) = MDE(t) − 1 · σ(5)

for the 90% tolerance interval:

S90(t) = MDE(t) + 10 · σ;  I90(t) = MDE(t) − 10 · σ(6)

where M(t)_i_—is the cumulative mass eroded at time t_i_, defined by the median curve (Rel. 1);

The experimental values of the standard deviations (σ) and the tolerance interval serve as indicators of several critical factors. Firstly, they assess whether the parameters of the treatment technology for sets of three samples were adhered to. Secondly, they gauge the correctness of the cavitation test procedures and whether all procedural steps were followed diligently. Furthermore, they evaluate the control and management of parameters influencing cavitation hydrodynamics, such as the constancy of its destructive intensity (including amplitude and frequency of vibrations, power of the electronic vibration generator, and water temperature). These parameters were meticulously monitored and controlled through software implemented in the computer, guiding the experimental process.

The data depicted in the diagrams from Figure 5, Figure 6 and Figure 7 underscore several key aspects:

- The precision of measurements was conducted on sets of three samples, with stringent control of parameters determining the intensity of vibrational cavitation. This precision is evidenced by the standard deviations (σ) ranging from 0.032 to 0.049 and the dispersion range of values corresponding to tolerance intervals of 95–96%, irrespective of the structural condition of the samples;
- Similar behaviors were exhibited by the three sets of samples, each corresponding to a distinct structural state, over the duration of cavitation exposure. This consistency indicates the accurate execution of the laser remelting technological process, with precise control of technological parameters such as scanning speed, laser beam diameter, and power. Consequently, similar hardness values are achieved on surfaces that are quickly remelted and solidified;
- The comparable evolution of median curves M(t) suggests that surfaces remelted under the three regimes exhibit very similar cavitation resistance and behaviors, with minor discrepancies in hardness values;
- Natural variations observed in the σ values imply that laser remelting at a speed of v = 1.2 m/min yields a microstructure that offers the most robust resistance to cavitation erosion.

There is a clear relationship between the differences between the three experimental values measured for each group of samples at the same cavitation attack times and the size (or mass) of grains released at the midpoints of the cavitation period. These differences are distinctly illustrated by the differences in the experimental data, expressed as an algebraic mean, when compared to the median curves v(t) displayed in Figure 8, Figure 9 and Figure 10.

The diagrams presented in Figure 8, Figure 9 and Figure 10 illustrate the median curves v(t) of experimental values for the mean erosion penetration rates corresponding to intermediate cavitation intervals, Δti. The data from these diagrams underscore several key observations:

- The v(t) curves exhibit similar trends, initially increasing towards a maximum and then experiencing a slight decrease towards the stabilized value vs. this pattern, consistent with previous experiences, suggests that structures and mechanical properties resulting from remelting have acquired high resistance to the impact of cavitation microjets;
- Very small differences between the experimental values, and in certain periods even identical values, reaffirm the conclusions drawn from the analysis of the data in Figure 5, Figure 6 and Figure 7. Specifically, they support the notion that each set of three samples from distinct structural states demonstrates approximately similar behaviors and resistances to vibrational cavitation solicitations throughout the attack duration.

Furthermore, insights into the differences between the resistances of structures determined by the laser scanning speed can be gleaned from the values of the ratios between the characteristic parameters M_max_ and vs, as defined by the curves M(t) and v(t):

(7) $$\frac{Mmax1}{Mmax3}=1035\;\;\;\;\;\;\;\;\;\frac{Vs1}{Vs3}=1.068$$

(8) $$\frac{Mmax2}{Mmax3}=1022\;\;\;\;\;\;\;\;\;\frac{Vs2}{vs3}=1.034$$

(9) $$\frac{Mmax1}{Mmax2}=1013\;\;\;\;\;\;\;\;\;\frac{Vs1}{Vs2}=1.033$$

where 1 corresponds to the scanning speed of 0.8 m/min, 2 corresponds to the scanning speed of 1.0 m/min, and 3 corresponds to the scanning speed of 1.2 m/min;

These values indicate the following:

- The increase in resistance achieved by remelting at a scanning speed of 1.2 m/min, compared to samples processed at laser scanning speeds of 0.8 m/min and 1.0 m/min, is slightly higher, ranging from 2.2% to 3.5% when analyzed based on the maximum value of the cumulative mean mass (M_max_), and from 3.4% to 6.8% when analyzed based on the value vs. toward which the mean erosion rate tends to stabilize;
- The increase in resistance achieved at a working speed of 1.0 m/min, compared to samples processed at a laser scanning speed of 0.8 m/min, is insignificant (only a 1.3% increase) when analyzed based on the maximum value of the cumulative mean mass (Mmax), and slightly higher (around 3.3%) when analyzed based on the value vs. toward which the mean erosion rate tends to stabilize.

In the graphs from Figure 11 and Figure 12, specific curves M(t) and v(t) for the four structural states are comparatively presented, utilizing the algebraic mean of the experimental values obtained from sets of three samples each (as shown in Figure 5, Figure 6, Figure 7, Figure 8, Figure 9 and Figure 10).

The data presented in the two diagrams reveal the following observations:

- Regardless of their type, whether M(t) or v(t), the trends of the mean curves and the level of experimental values, represented by the algebraic mean, indicate that surfaces hardened by laser remelting exhibit significantly superior behavior compared to those treated with bulk homogeneous annealing heat treatment;
- The values of standard deviations (σ) show that the dispersions of the experimental values obtained on samples hardened by laser surface remelting are significantly smaller than those obtained with homogeneous annealing.

No matter the scanning speed, the increase in structural resistance to the destructive impacts of cavitation microjets obtained from laser remelting, when compared to the annealed condition, ranges from 4.9 to 5.1 times based on the maximum values of mass losses (defined by the median curves), and from 4.6 to 5 times based on the mean erosion rates values (v_s_). An essential indicator in assessing the resistance of a structure to the destructive effects of cavitation microjets, recommended by ASTM G32-2016, is the cavitation resistance (R_cav_), defined as the inverse of the erosion rate value toward which the v(t) curve tends to stabilize. In our case, this value is observed at 165 min.

The values of this parameter are as follows:

(10) $$R_{\mathrm{cav}}v=0.8\;m/\min=\frac{1}{0.031}=32.25\;\min/\mathrm{mg}$$

(11) $$R_{\mathrm{cav}}v=1\;m/\min=\frac{1}{0.03}=33.33\;\min/\mathrm{mg}$$

(12) $$R_{\mathrm{cav}}v=1.2\;m/\min=\frac{1}{0.029}=34.48\;\min/\mathrm{mg}$$

(13) $$R_{\mathrm{cav}}\mathrm{Annealing}=\frac{1}{0.145}=6.85\;\min/\mathrm{mg}$$

From these values, it is evident that hardening with a laser beam results in significantly higher resistances (4.7 to 5 times) to cavitation erosion compared to the bulk homogeneous annealing heat treatment. This substantial improvement in resistance underscores the effectiveness of laser remelting in enhancing the structural integrity and durability of the material against the destructive effects of cavitation microjets.

### 3.2. Microstructural Examinations

**Figure 13:**
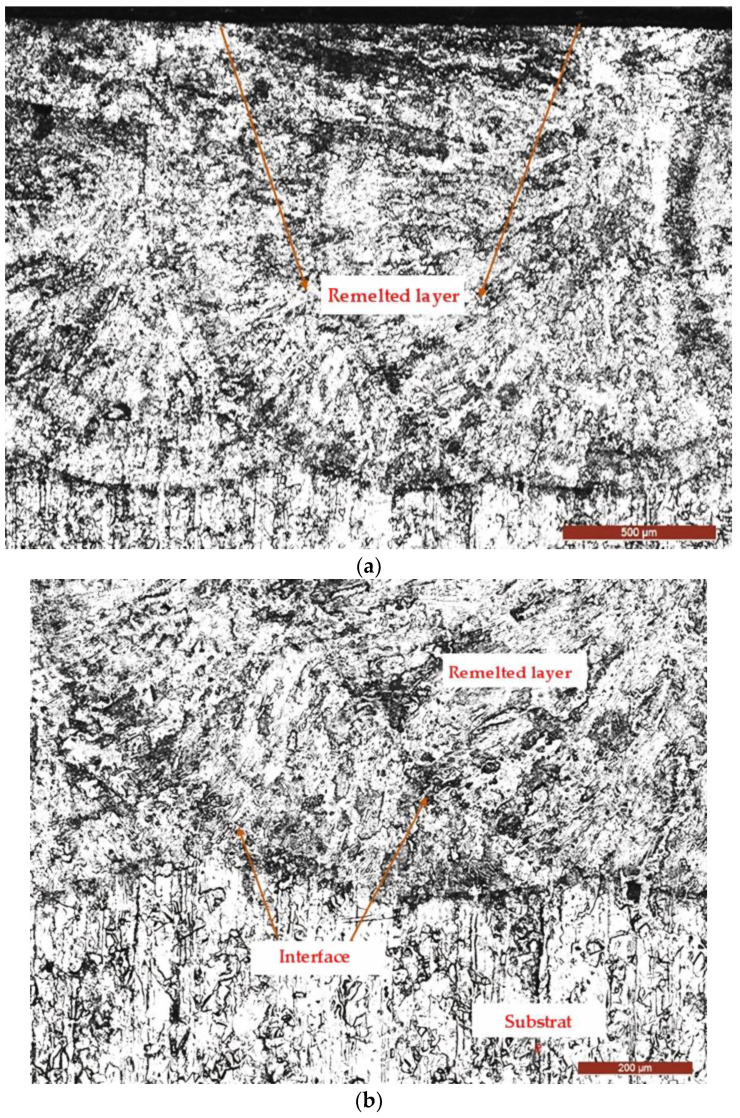

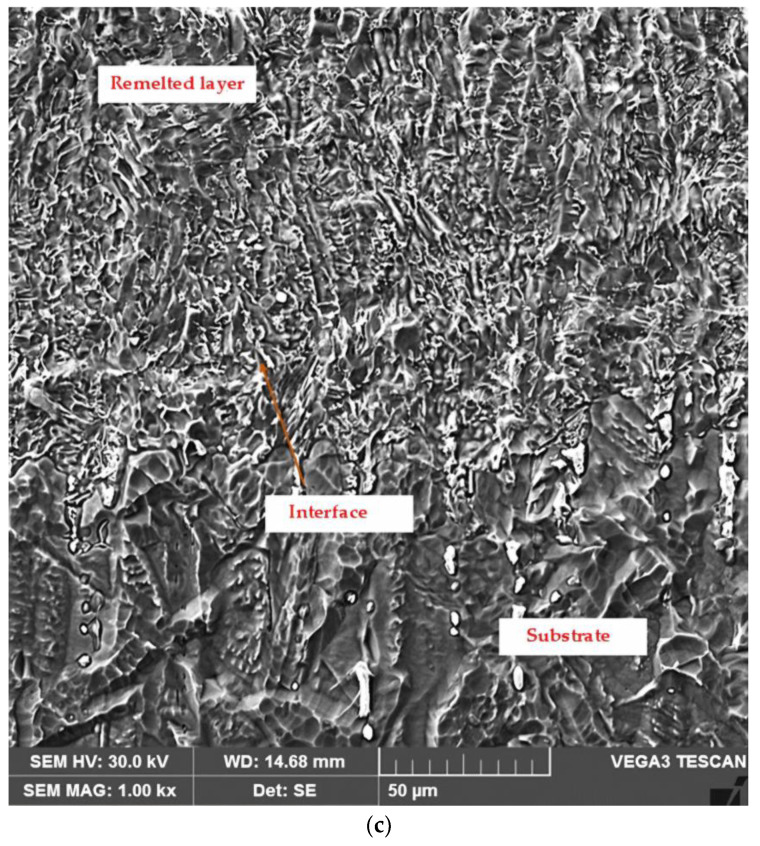
The microstructure of the coating–substrate system: (**a**)—× 50; (**b**)—× 100; (**c**)—SEM × 1000.

**Figure 14:**
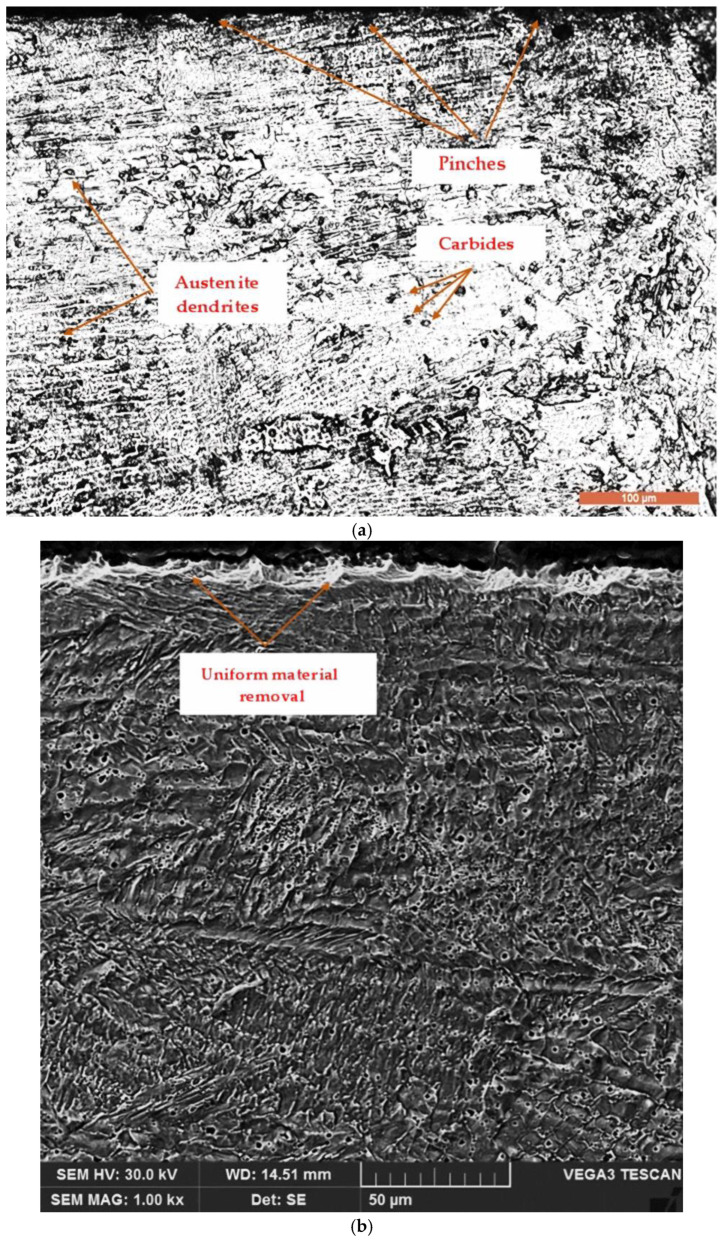
The microstructure of the layer tested under cavitation for 165 min: (**a**)—OM × 200; (**b**)—SEM × 1000.

Some of the samples subjected to cavitation erosion tests underwent conventional metallographic techniques, including grinding, polishing, and chemical etching, for microstructural examinations. Figure 13 and Figure 14 present representative images illustrating the degradation of the surface layer microstructure.

These images demonstrate that the degradation observed has a predominantly mechanical nature and is characterized by the cyclic impact of the surface by high-speed cavitation microjets generated by the implosion of bubbles formed through the mechanism of vibratory cavitation. The microstructure of the laser-treated layer appears fine and uniform, with hardness values ranging from 320 to 390 HV5, significantly higher than those of the reference material (205 to 215 HV5). The highest hardness values are specific to surface remelting at a scanning speed of 1200 mm/min, and these correspond to the lowest erosion rates. This phenomenon is explained by the rapid cooling of the molten metal bath, which increases the degree of undercooling, leading to a shift toward lower solidification temperatures. Additionally, increased undercooling reduces the crystallization of the nucleus size and the energy required for its formation.

The refinement of grain structure and limitation of secondary phase precipitations following laser surface remelting enhance the material’s ability to absorb the energy of cavitation impact waves, which delays crack nucleation.

From Figure 14, it can be observed that the degradation of the surface tested for cavitation over 165 min is relatively uniform, and the initiation of cracking primarily occurs at the boundaries between austenite grains and precipitated chemical compound particles from the melt and austenite.

### 3.3. X-Ray Diffraction Analyses

**Figure 15:**
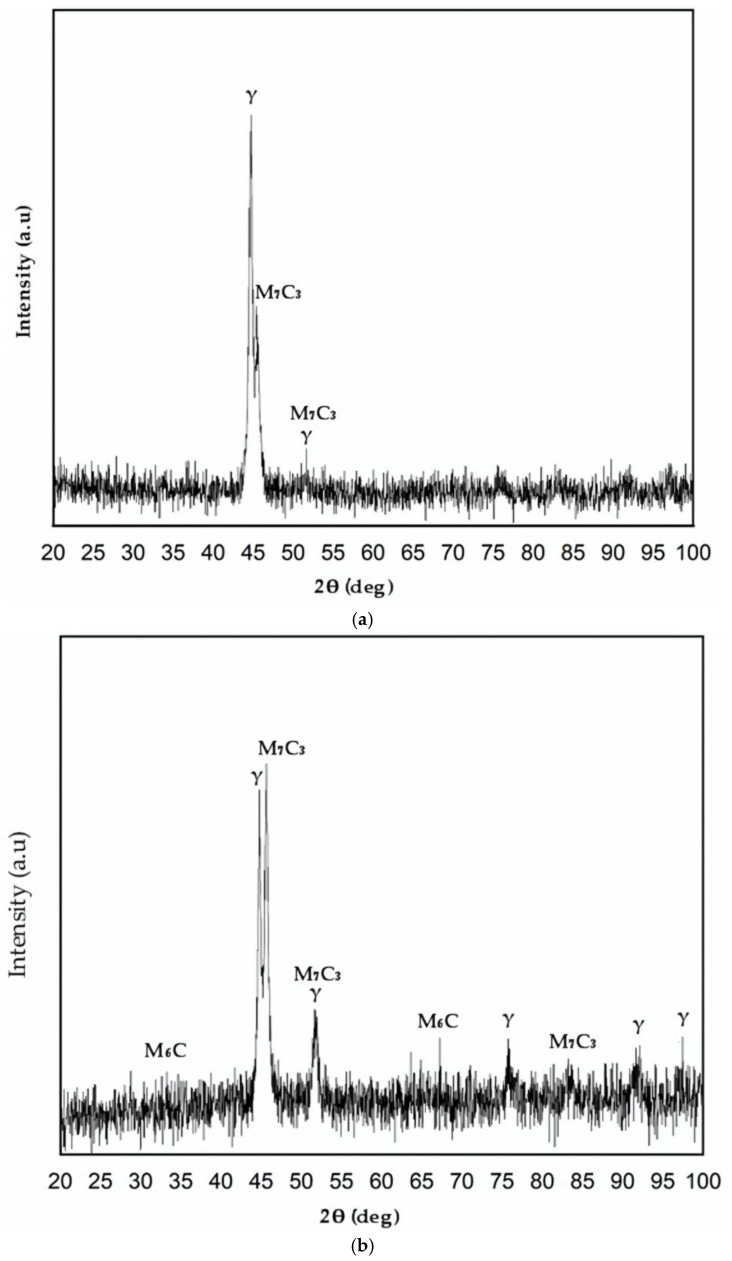
X-ray diffraction patterns for reference material (**a**) and laser-treated surface (**b**).

The reference metal and the laser-treated surface layers were subjected to X-ray diffraction examinations using a Philips X’Pert diffractometer fitted with a graphite monochromator for Cu-Kα radiation (λ = 1.54 Å) at room temperature. At a rate of 1°/min, measurements were made in 2 theta geometry between 20° and 100°. The process was carried out with a voltage of 40 kV and a current intensity of 30 mA. Crystallographic identification of the phases in the samples was performed using the Joint Committee on Powder Diffraction Standards (JCPDS) database. Figure 15 presents the diffraction patterns obtained from the reference material and the laser-treated surface layer.

The dominant phase in the unaffected base metal is γ-iron, and carbide peaks M_7_C_3_ and M_6_C appear in the melted surface layer along with γ-iron, as can be observed by comparing the position angles of the interference peaks with standard ones for ferrite, martensite, austenite, and carbides. These results are in concordance with previous investigations carried out by other researchers [31,32].

### 3.4. Corrosion Test Results

Electrochemical corrosion measurements were conducted in a saline environment with a sodium chloride solution (3.5% NaCl) and an accelerated corrosion environment containing a 0.5 M H_2_SO_4_ solution, following the ASTM G5-2014 standard [37]. The surface area of the specimen in contact with the corrosive medium was 1 cm^2^. Polarization curves were plotted for each sample and testing condition. By drawing tangents between the cathodic and anodic sections, electrochemical parameters such as corrosion potential and current density were determined.

**Figure 16:**
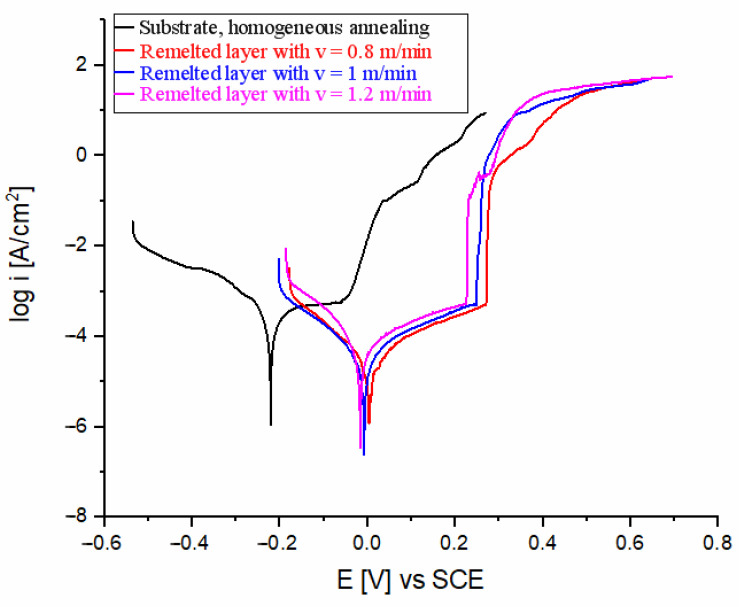
Polarization curves in 3.5% NaCl solution.

**Figure 17:**
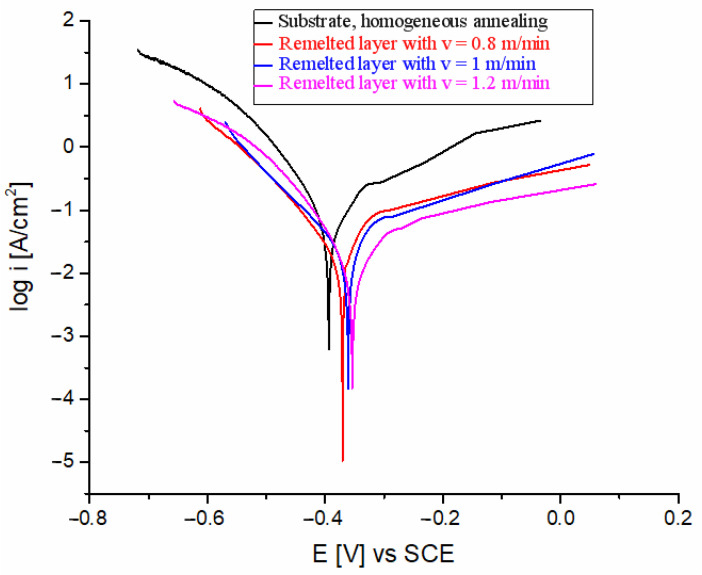
Polarization curves in 0.5 M H_2_SO_4_ solution.

In Figure 16 and Figure 17, comparative polarization curves of the investigated steel before and after laser beam remelting are presented in the two corrosive test environments. Tafel polarization curves provide insights into anodic and cathodic reactions. The anodic branch represents the material’s oxidation (metal dissolution), while the cathodic branch reflects the reduction reactions (typically oxygen reduction or hydrogen evolution). From the analysis of the electrochemical data presented in Table 2 and Table 3, it is noticeable that laser beam remelting had a positive effect on corrosion behavior compared to the reference material (substrate). It is known that better corrosion properties, often indicated by lower current densities during electrochemical testing, are associated with reduced rates of material degradation in corrosive environments [38].

When testing the samples in a 3.5% NaCl saline solution (Figure 16), a good performance of stainless steel is observed regardless of the laser beam scanning speed. From Table 2, it can be seen that the corrosion current values decrease from approximately 0.181 µA/cm^2^ (reference material/substrate) to about 0.023–0.096 µA/cm^2^, indicating an increase in electrochemical corrosion resistance by 4.4–6.3 times. The grain refinement achieved by the laser remelting process improved the corrosion resistance due to the ability of the fine-grained material to passivate better. The remelted samples showed a more positive E_corr_ and a lower anodic slope compared with the substrate due to effective passivation.

**Figure 18:**
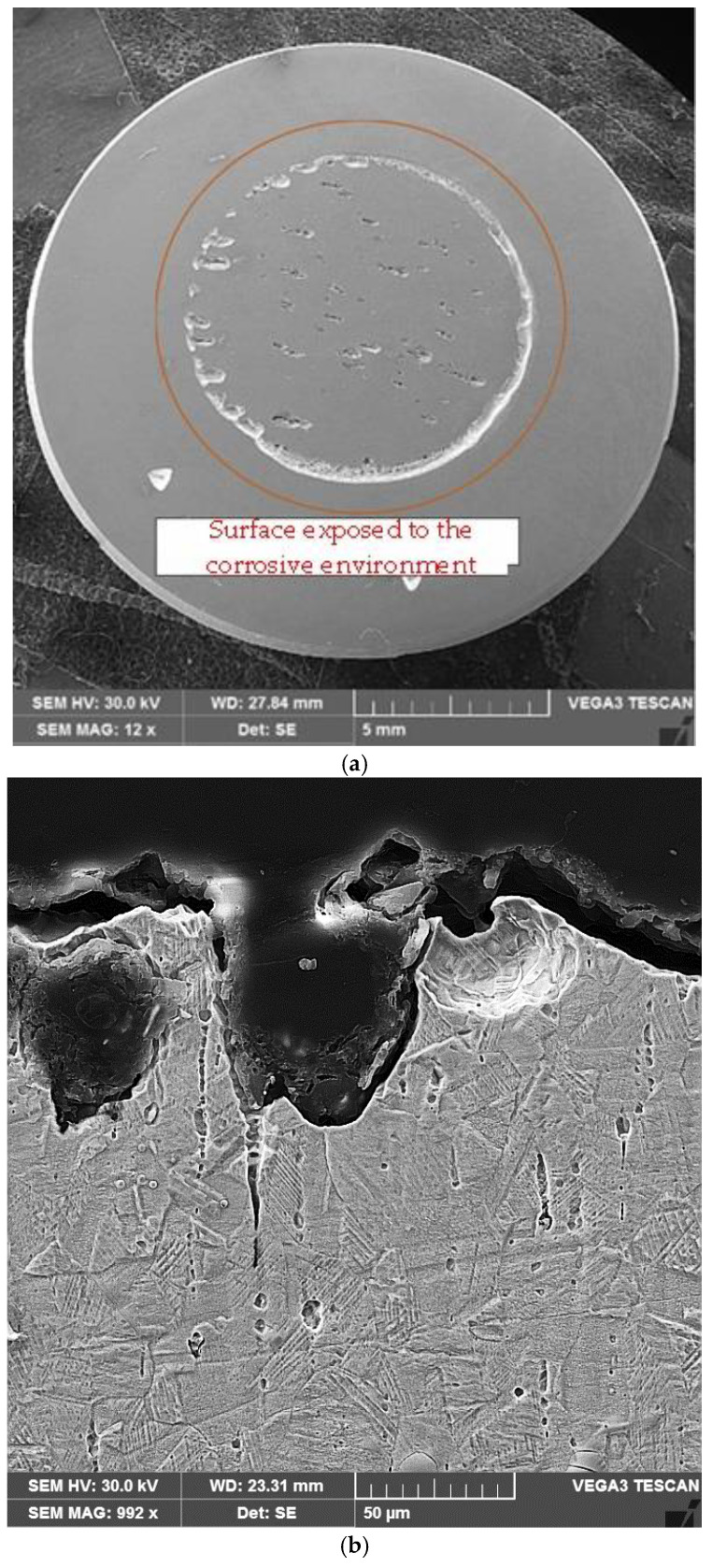

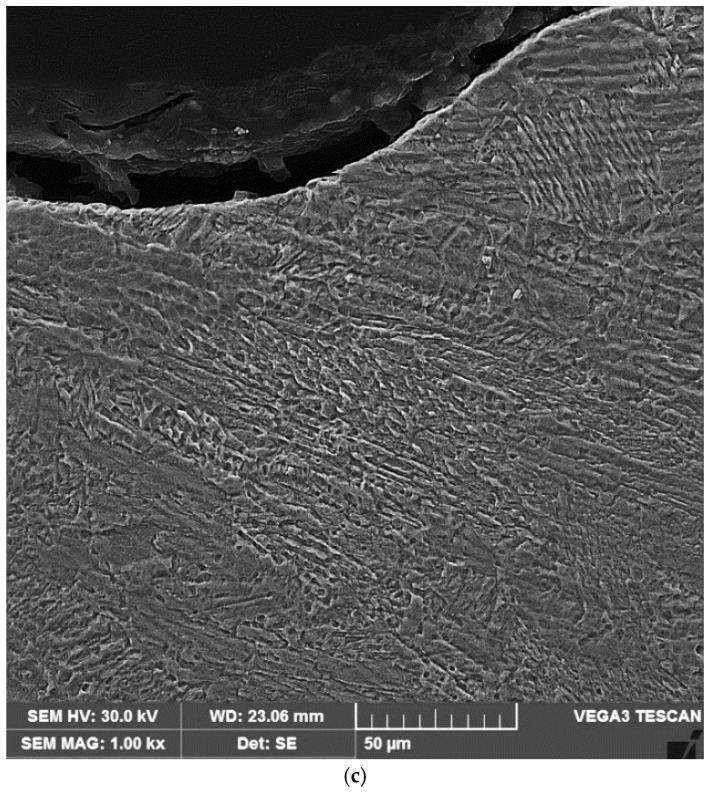
Local corrosion degradation of the material in samples exposed to 0.5 M H_2_SO_4_ solution: (**a**) surface of the corroded area; (**b**) cross-section of the corroded zone of the substrate; (**c**) cross-section of the laser-remelted layer.

The surface exposed to the corrosive environment (marked with the orange circle) after testing in the salt solution did not exhibit macroscopic signs of corrosion. In the case of the 0.5 M H_2_SO_4_ acidic solution, the corrosion phenomenon manifested differently, and material degradation occurred rapidly. All samples exposed to the acidic environment showed weaker corrosion resistance (Figure 17), with the corrosion current (Table 3) increasing up to values of 39.52 µA/cm^2^ (reference sample/substrate). On the surface exposed to the corrosive environment, degraded areas in the form of pits and large craters with intergranular regions connecting them were formed (Figure 18).

The results indicate that in both environments, the samples remelted at a scanning speed of 1200 mm/min exhibited the best corrosion behavior. Laser surface treatment under this regime produced a homogeneous and refined microstructure, free from carbide precipitation, with fine granulation that enhanced the material’s chemical properties.

Nevertheless, the laser-remelted samples exhibited a slightly improved corrosion behavior compared to the reference sample (Table 3), with the corrosion current decreasing to values of up to 5.24–9.12 µA/cm^2^. In the substrate microstructure, the corrosive media creates defects that provoke the degradation of the material [38].

## 4. Conclusions

Based on the experimental data obtained, it can be concluded that the technique of local laser beam surface remelting offers the following advantages:

- Significantly improves resistance to cavitation erosion and electrochemical corrosion;
- Develops a metallurgical bond at the interface between the layer and the substrate, resulting in enhanced adhesion of the coating;
- Refines the microstructure and increases the hardness of the coating layer;
- Provides greater flexibility, such as shorter processing time, fewer thermal distortions, and minimal changes to the substrate’s microstructure.

Future research aims to establish a correlation between the influencing factors derived from fractal analysis, the electrochemical impedance spectroscopy (EIS) test, and the specific parameters of cavitation erosion recommended by ASTM G32-2016.

## Figures and Tables

**Figure 11:**
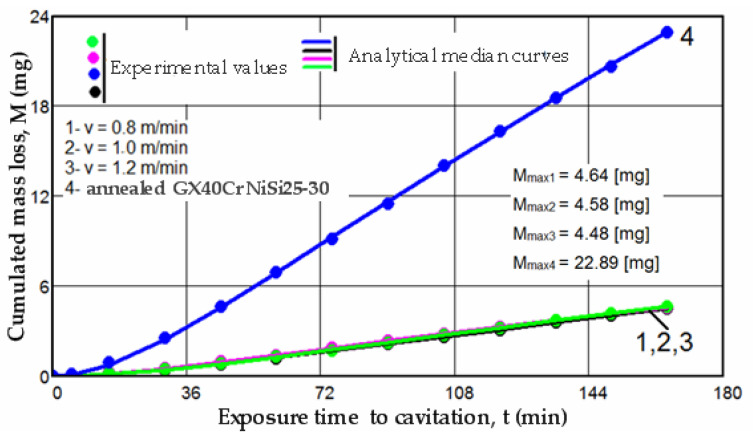
Comparison based on cumulated mean mass losses.

**Figure 12:**
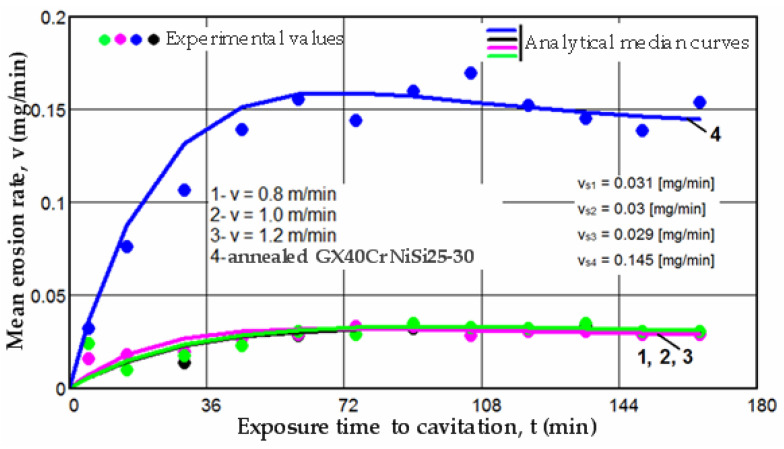
Comparison based on the curves of mean erosion rates.

**Table 1:** GX40CrNiSi25-20 steel chemical composition, wt%.

Carbon (C)	0.3–0.5
Silicon (Si)	1–2.5
Manganese (Mn)	max. 2
Nickel (Ni)	19–22
Phosphorous (P)	max. 0.04
Sulfur	max 0.03
Chromium	24–27
Molybden (Mo)	max. 0.5
Iron (Fe)	Balance

**Table 2:** Corrosion data of the tested samples in chloride solution (3.5% NaCl).

Sample	E [mV]	i_corr_ [µA/cm^2^]
Substrate, homogeneous annealing	−219	0.181
Remelted layer with v = 0.8 m/min	5.69	0.023
Remelted layer with v = 1.0 m/min	−14.4	0.096
Remelted layer with v = 1.2 m/min	−7.62	0.041

**Table 3:** Corrosion data of the tested samples in acid solution (0.5 M H_2_SO_4_).

Sample	E [mV]	i_corr_ [µA/cm^2^]
Substrate, homogeneous annealing	−403.7	39.52
Remelted layer with v = 0.8 m/min	−370.23	9.12
Remelted layer with v = 1.0 m/min	−360.15	8.76
Remelted layer with v = 1.2 m/min	−352.47	5.24

## Data Availability

Data are contained within the article.
